# Covariant description of X-ray diffraction from anisotropically relaxed epitaxial structures

**DOI:** 10.1107/S0021889813006171

**Published:** 2013-06-07

**Authors:** A. Zhylik, A. Benediktovitch, I. Feranchuk, K. Inaba, A. Mikhalychev, A. Ulyanenkov

**Affiliations:** a Belarusian State University, Minsk, Belarus; b X-ray Research Laboratory, Rigaku Corporation, Akishima, Tokyo, Japan; c Rigaku Europe SE, Ettlingen, Germany

**Keywords:** epitaxial layers, relaxation parameters, high-resolution X-ray diffraction, theoretical approach

## Abstract

A general theoretical approach to the description of epitaxial layers with essentially different cell parameters and in-plane relaxation anisotropy has been developed. A covariant description of relaxation in such structures has been introduced. An iteration method for evaluation of these parameters on the basis of the diffraction data set has been worked out together with error analysis and reliability checking. The validity of the presented theoretical approaches has been proved with *a*-ZnO on *r*-sapphire samples grown in the temperature range from 573 K up to 1073 K. A covariant description of relaxation anisotropy for these samples has been estimated with data measured for different directions of the diffraction plane relative to the sample surface.

## Abstract 2

A general theoretical approach to the description of epitaxial layers with essentially different cell parameters and in-plane relaxation anisotropy is presented.

## 1. Introduction

High-resolution X-ray diffraction (HRXRD) is one of the most effective tools for investigation and nondestructive control of epitaxial crystalline thin-film layers (Pietsch *et al.*, 2004 ▶). An important part of HRXRD analysis is defined by construction of the appropriate sample model and connection of its parameters with diffraction pattern features. It is well known that the interaction between atoms in adjacent thin films leads to deformation of the crystal unit cells that depends on the layer thickness. In the case of layers having a cubic crystallographic system and being isotropic, in-plane characterization of this deformation is a well established procedure with the relaxation *R* as the only parameter connected with in-plane strain of the unit cell (mismatch). However, nowadays epitaxial layers with hexagonal materials in different orientations are widely used in industrial light-emitting diode production (Paskova, 2008 ▶). In this investigation, a complicated epitaxial relation in combination with an in-plane anisotropy appear (Laskar *et al.*, 2011 ▶). Consequently more parameters should be used for the accurate characterization of unit-cell deformations. In the present paper, a set of parameters allowing one to describe layers with anisotropic epitaxial relations is introduced and a general procedure for evaluation of these parameters is developed, based on the X-ray diffraction profiles or reciprocal space maps (RSM). We consider this a covariant description because it can be used in the same form for arbitrary symmetry and orientation of the crystal unit cells in each layer. A series of papers (Yang *et al.*, 1994 ▶; Caro & Tapfer, 1995 ▶, and references therein; Caro *et al.*, 1996 ▶; Bottomley *et al.*, 2001 ▶; Brandt *et al.*, 2002 ▶) have been devoted to theoretical analysis of similar problems. However, in these works the fully pseudomorphic layers were considered and relaxation anisotropy was not taken into account.

We are proposing a generalized theoretical approach to describe the relationships between multiple crystalline layers that may display relative mismatches and in-plane anisotropy. The validity of XRD data evaluation was verified on the basis of an iteration scheme for error analysis and reliability checking. Diffraction data were collected in three different crystallographic directions from *a*-ZnO on *r*-sapphire samples grown at different temperatures from 573 to 1073 K. The analysis was performed according to the approach described above.

The paper is organized as follows. In *Theoretical approach* [Sec sec2], we show how the conventional parameters used for the description of the epitaxial layer state for cubic in-plane isotropic layers can be generalized for in-plane anisotropic layers with arbitrary epitaxial relations. In *Fit algorithm and error analysis* [Sec sec3], we describe a way to get values and errors of sample parameters from Bragg peak positions obtained from the set of diffraction profiles/RSM to describe layers with arbitrary epitaxial relations. Application to *a*-ZnO films on *r*-sapphire substrates, grown at different temperatures from 573 to 1073 K, is presented in *Experimental: a-ZnO on r-sapphire* [Sec sec4].

## 2. Theoretical approach

**Figure 1:**
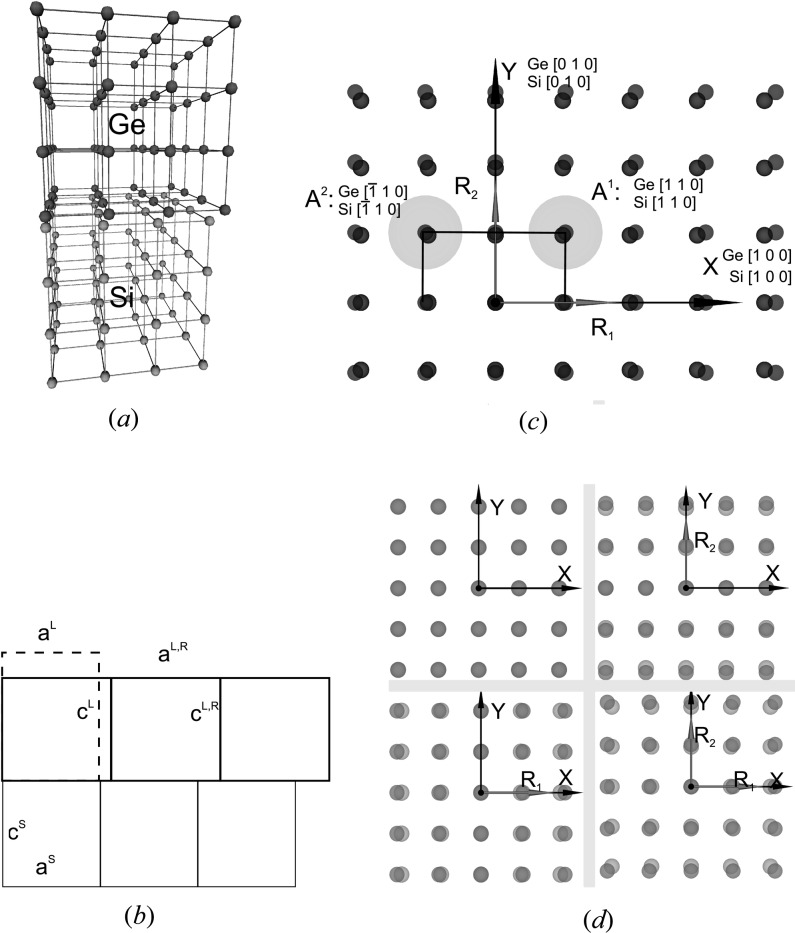
Models of partly relaxed epitaxial Ge(001) layers on Si(001) substrates. (*a*) Crystal lattices of a relaxed Ge(001) layer on an Si(001) substrate. (*b*) Conventional relations of a Ge(001) epitaxial layer on Si(001). *a* ^S^ and *c* ^S^ are the lattice constants of the bottom Si(001) layer. 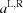 and 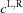 are the relaxed lattice constants of the top Ge(001) layer. *a* ^L^ and *c* ^L^ are the actual or strained lattice constants of the top Ge(001) layer. (*c*) Coincidence-site lattices of a Ge(001) epitaxial layer on Si(001). Axes *X* and *Y* correspond to sample basis axes **S**_1_ and **S**_2_. Relaxation vectors **R**_1_ and **R**_2_ have directions 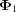 and 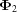 of the sample principal basis and length values corresponding to relaxation degree. Gray circles mark out coinciding pairs of lattice nodes – anchors *A* ^1^{Ge[110]; Si[110]}, *A* ^2^{Ge[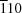]; Si[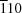]}. (*d*) Demonstration of anisotropic relaxation of an epitaxial layer. Relaxation vectors **R**_1_ and **R**_2_ show the directions of relaxation and strain degree.

Here we provide a reminder of the principal definitions of the conventional approach for the cubic crystallographic system (Fig. 1 ▶ *a*) and isotropic in-plane relaxation. The typical crystallographic representation of the interface between two epitaxial layers is displayed in Fig. 1 ▶(*b*). Using the lattice constant values as a basis for further definitions, the lattice constant mismatch *F* of the relaxed top layer or initial mismatch is defined as follows:
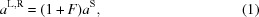
where 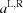 is the lattice constant for the relaxed layer. In this paper, we will consider only the strain of the pseudomorphic layer so the structure of the relaxed layer and the parameter *F* are supposed as known values. It is also assumed that the other in-plane lattice constant 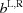 is defined utilizing the same equation (isotropic relaxation).

The difference between the relaxed and the actual lattice constants is characterized by the relaxation degree *R*, 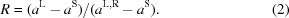
This is closely connected with the actual mismatch of the top layer, which is usually defined with the diffraction data, 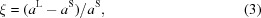
and its strain 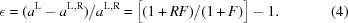
The out-of-plane lattice constant *c* can be found on the basis of Hook’s law, which is expressed as the Poisson ratio for the considered isotropic case: 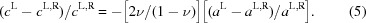



In order to generalize equations (1)–(5) [Disp-formula fd3] [Disp-formula fd4] [Disp-formula fd5] for anisotropic relaxation (two-dimensional) we should introduce a ‘sample’ orthogonal coordinate system *S*: **S**_1_, **S**_2_, **S**_3_ with **S**_3_ parallel to the sample normal and **S**_1_, **S**_2_ describing the interface plane. There is some degree of freedom for selection of **S**_1,2_ that will be used below.

Let us assume that the pseudomorphic state interface between layer and substrate has an in-plane translation symmetry, forming a coincidence-site lattice (CSL) (Zur & McGill, 1984 ▶), which is in general different from those of layer and substrate. The actual in-plane translation symmetry of the CSL can be parameterized by defining three nodes in the layer and in the substrate which coincide. We will call them anchors. The anchors are given in Miller indices in crystallographic reference systems for the layer *C*_L_ and for the substrate *C*_S_; hence they enable the description of the mutual arrangement of *C*_L_, *C*_S_ and *S*. One of the anchors is by definition [000] in both *C*_L_ and *C*_S_. The other two anchors are in general given by the sum of CSL translation vectors with integer factors.

For example, the trivial case of relaxed Ge on Si in terms of anchors can be described in the following way:
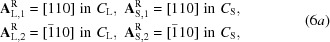
or by
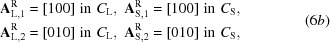
both leading to the same CSL. Compared with the epitaxial relations, anchors define not only the directions of CSL translation vectors but their magnitude as well.

In order to proceed with the generalization of equations (1)–(5) [Disp-formula fd3] [Disp-formula fd4] [Disp-formula fd5] let us introduce the following quantities:
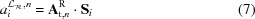
for the relaxed top layer anchor projections on the sample axis, 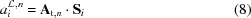
for the actual top layer anchor projections on the sample axis, and
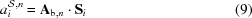
for the bottom layer (substrate) anchor projections on the sample axis; the anchor projections on **S**_3_ are zero by definition.

Then the mismatch between the relaxed layer and the substrate is described by the known 2 × 2 matrix 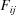 [compare with equation (1)]: 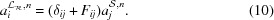
The matrix 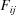 describes the strain only; the net rotation is supposed to be excluded from the transformation (10), therefore 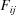 is a symmetrical matrix. The vectors **S**_1_, **S**_2_ can be chosen as the principal axes **Φ**_1_, **Φ**_2_ of 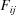. In this in-plane coordinate system, 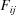 has a diagonal shape and can be described by two eigenvalues 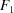, 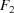: 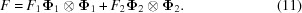
The actual mismatch matrix 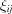 connects the anchor projections in the following way [compare with equation (3)]: 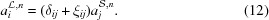
The relaxation matrix 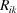 connects the actual mismatch 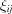 and the initial mismatch 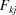 as follows: 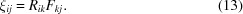
In this coordinate system 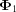, 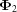 the relaxation tensor will follow the strain tensor and have a diagonal form. It is described by two values, 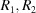, that can be considered as the covariant relaxation parameters. Consequently the actual mismatch tensor can be expressed in the following way: 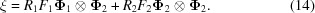
The in-plane strain tensor 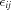 of the top layer connects the projections of the actual top layer and the relaxed top layer: 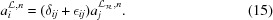
The strain can also be expressed through the relaxation and the initial mismatch [compare with equation (4)]: 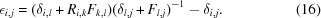



In order to reconstruct a complete deformation status of the crystallographic unit cell, all the components of the three-dimensional strain tensor have to be calculated. Hook’s law, linking the strains and the stresses by the stiffness tensor, is used: 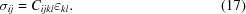
Taking into account that the in-plane strain tensor components 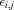 are known, the strain and the stress tensors are symmetric, and the vertical components of the stress tensor equal zero because only in-plane forces σ_13_ = σ_23_ = σ_33_ = 0 exist, the values 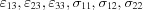 can be found from equation (17). As the result, the complete strain tensor is obtained: 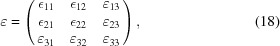
where the components 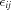 are defined by relaxation parameters (16) and 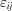 are calculated from equation (2).

Knowing the three-dimensional strain, it is straightforward to find the parameters of the strained crystallographic cell. The connection of the crystallographic cell basis **e**_1_, **e**_2_, **e**_3_ with the cell parameters is 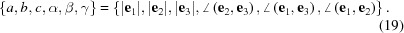



The decomposition of the relaxed crystallographic cell basis vectors in the principal axes basis of the initial mismatch is 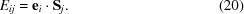
Hence, the decomposition of the strained crystallographic cell basis vectors in the principal axes basis of the initial mismatch is given by 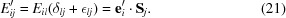
Finally, the parameters of the strained crystallographic cell are 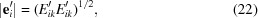


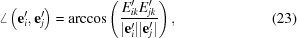
where in equation (22) summing over the index *i* is not performed.

The proposed method is valid for every crystallographic system. It can be shown that the expressions for strain and unit-cell deformation, as obtained from Romanov *et al.* (2006 ▶) and Laskar *et al.* (2011 ▶) for specific cell configurations, can be derived from the equations above.

## 3. Fit algorithm and error analysis

Once the parameters from equation (20) are known, the direct diffraction problem can be solved, *i.e.* the positions of Bragg peaks in diffraction profiles and/or RSM can be calculated for an arbitrary sample. However, in most cases the solution of the inverse problem, that is the evaluation of the sample parameters (like solid solution concentration, relaxations 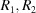 *etc*.) from a series of Bragg peak positions, is required. In the one-dimensional case this problem can be solved analytically. In the considered two-dimensional case, a quite large system of linear equations has to be solved. Following from equations (7)–(23) this should be carried out for each set of available diffraction data, *e.g.* symmetric and asymmetric profiles, or RSM, *etc*. Direct solution of these equations is not effective because the experimental data could be limited or, in the opposite case, the system of equations could be overcomplete. Therefore we have evaluated these parameters by fitting all experimental Bragg peak positions that could be extracted from the diffraction profiles and RSM in a concrete experiment. The proximity of the measured Bragg peak position to the theoretical one has been used as a partial cost function 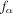 which should be equal to zero for exact values of the sample parameters.

The actual form of the cost function 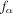 depends on the experimental setup. If an open detector is used, the Bragg peak position in a diffraction profile corresponds to the direction of the incoming wavevector **k**_in_ which satisfies the Bragg condition **H** ^(S)^ = **k**_out_ − **k**_in_, while the direction of the outgoing wavevector **k**_out_ cannot be obtained owing to the large detector aperture. In this case, we have used the following cost function:
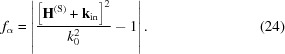
Here **H** ^(S)^ is the reciprocal lattice vector calculated on the basis of sample parameters of interest from equations (7)–(23).

In the case of RSM, both **k**_in_ and **k**_out_ can be found from the scattering experiment and the cost function is defined as 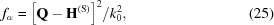
where **Q** = **k**_out_ − **k**_in_ is the transferred wavevector.

The cost function from a single peak provides only some link between parameters, *e.g.* the peak position in a symmetric scan can give only the connection between relaxation and concentration. To determine each of them an additional asymmetric scan is needed. For an arbitrary set of diffraction data we have built up the complete cost function 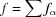. Then the parameters were evaluated by means of minimization of this cost function.

In order to find out whether the parameters of interest can be obtained from a given set of diffraction data, a procedure for evaluation of their errors has been developed. Generally, some information for the estimation of the errors can be gained from the analysis of the optimization algorithm convergence. In the case of fitting overcomplete sets of measured data this kind of error estimation can be sufficiently accurate, because of the random character of the experimental errors of different points in the considered data set (Giacovazzo *et al.*, 2002 ▶). However, when the number of the characteristic parameters of the measured data has the same order as the number of optimized sample parameters, the error estimation on the basis of the cost function can be incorrect. For example, when the numbers of the data and sample parameters coincide, the optimization can be made perfectly with the achieved value of the cost function being exactly equal to zero. However, the obtained results may still contain errors; they are just undetected by this method. This situation, commonly encountered in the problem of cell parameter determination from Bragg peak positions in set of diffraction profiles, is analyzed below.

Consider the set of normalized sample parameters 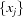, 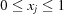 (like concentration, relaxation *etc*.), which are determined on the basis of fitting the set of parameters 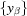 of the measured data (like Bragg peak positions) that are defined with some experimental errors 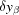 assumed to be independent. The correct values 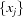 of the sample parameters correspond to the accurate values *y*_β_, containing no experimental errors, and are determined by minimizing the cost function 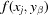: 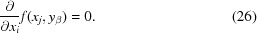
The values 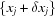 determined from the real (inaccurate) data are connected in the same way to the measured values 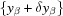: 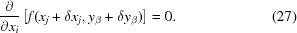



Expanding equation (27) to the first order of the errors and taking into account equation (26), we obtain the following equation for the inaccuracies 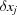: 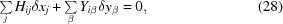
where 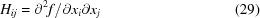
is the cost function Hessian for the sample parameters and
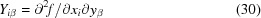
is the matrix of the mixed derivatives.

If the Hessian 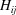 is a nonsingular matrix, equation (28) provides all the necessary information for estimation of the errors 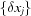: 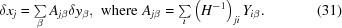
The deviations 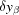 of the measured data from the exact values are unknown (otherwise the exact value could be found) and random in nature. From the experimental conditions and the methods of data processing, only the variances of the quantities 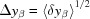 can be estimated. Taking into account the independence of the random variables, the following expression for the mean squared errors of the estimated sample parameters is found: 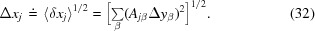
A more general consideration, accounting for both singular and nonsingular Hessians, is provided in Appendix *A* [App appa].

## 4. Experimental: *a*-ZnO on *r*-sapphire

**Figure 2:**
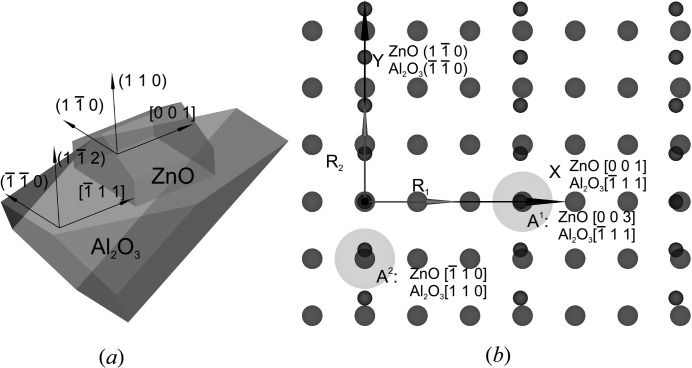
Sketch of an *a*-ZnO epitaxial layer on *r*-sapphire. (*a*) Mutual orientation of crystallographic cells of an *a*-ZnO heteroepitaxial layer on *r*-sapphire substrate. (*b*) Coincidence-site lattices of an interface ZnO(110) epitaxial layer on Al_2_O_3_(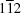) with anchors *A* ^1^{ZnO[003]; Al_2_O_3_[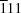]}, *A* ^2^{ZnO[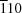]; Al_2_O_3_[110]} of *a*-ZnO on *r*-sapphire.

**Figure 3:**
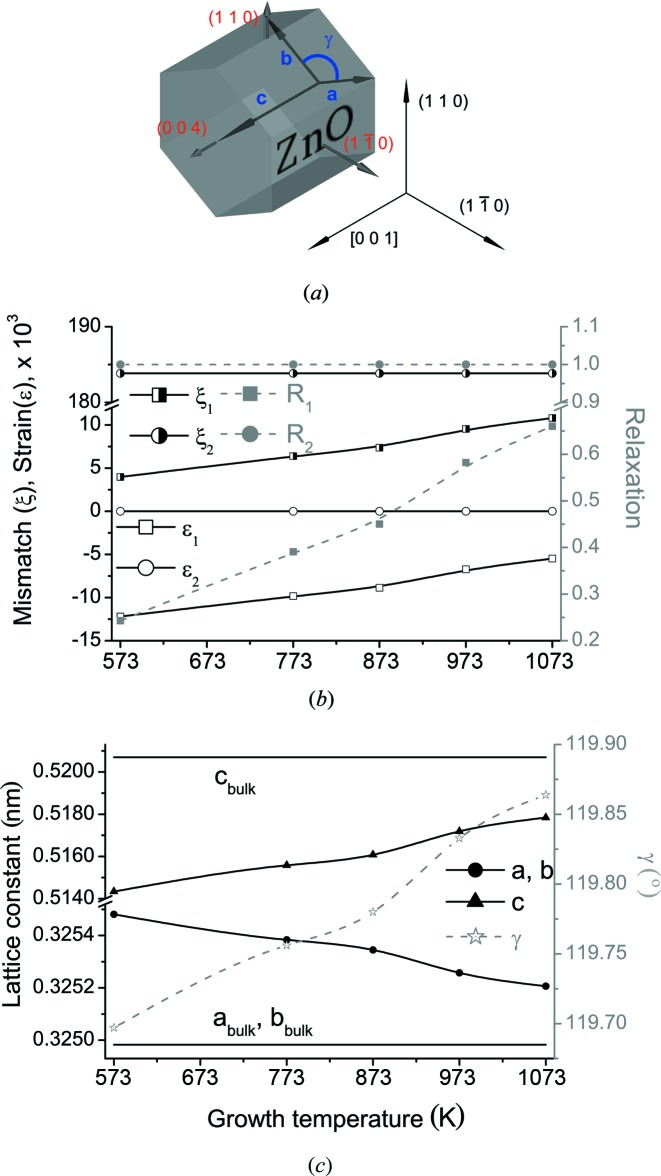
Lattice parameter evaluation of an *a*-ZnO layer on *r*-sapphire. (*a*) Measured reflections 004, 110 and 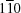 and crystallographic axes **a**, **b** and **c** of an *a*-ZnO heteroepitaxial layer on *r*-sapphire substrate. (*b*) Dependency of relaxations 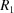, *R*_2_, strains 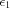, 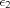 and mismatches 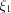, 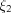 in two azimuthal directions [001] and 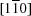 of an *a*-ZnO heteroepitaxial layer on *r*-sapphire on growth temperature. (*c*) Dependency of crystallographic cell parameters (*a, b, c*, γ) of an *a*-ZnO heteroepitaxial layer on *r*-sapphire on growth temperature. Bulk (relaxed) cell parameters (*a, b, c*) of ZnO are shown for eye guidance.

A set of *a*-ZnO films were grown on *r*-Al_2_O_2_ substrates. ZnO films were grown in the temperature range of 573–1073 K with an interval of 100 K. The ZnO film thickness was about 280 nm. Structural characterization for the *a*-ZnO/*r*-Al_2_O_3_ nonpolar heterostructures was performed with a Rigaku five-circle SmartLab system, equipped with a high-power X-ray source (45 kV, 200 mA) and a Bartels-type Ge(220) monochromator. For each sample, HRXRD measurements were performed at 110, 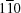 and 004 reflections (see Fig. 3*a*). The mutual orientation of the *a*-ZnO crystallographic cell on *r*-sapphire is presented in Fig. 2 ▶(*a*). According to epitaxial relations referred to in the paper by Han *et al.* (2012 ▶), pairs of anchors were specified as {ZnO[003]; Al_2_O_3_ 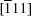} and {ZnO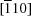; Al_2_O_3_[110]} (see Fig. 2 ▶ *b*). Relaxation parameters were found using the fitting algorithm with errors that are presented in Table 1 ▶. Strain and mismatch parameters were calculated from the relaxation. Relaxation, strain and mismatch results are shown in Fig. 3 ▶(*b*). Crystallographic lattice constants, including angle γ distortion of the ZnO layer, are calculated according to the proposed theoretical approach and the results are shown in Fig. 3 ▶(*c*).

## 5. Conclusion

A general formalism for describing anisotropic relaxation in layers with arbitrary epitaxial relations has been proposed. An iteration scheme of layer parameter determination from peaks of arbitrary sets of one- and two-dimensional diffraction data was considered. It is based on construction of a cost function and its minimization over layer parameters. Error evaluation and reliability checking of the obtained results has been described. Application of the proposed approaches has been demonstrated on X-ray diffraction data of *a*-ZnO on *r*-sapphire samples grown in the temperature range from 573 K up to 1073 K.

## Figures and Tables

**Table 1:** Dependency of relaxations *R*_1_ and *R*_2_ in directions [001] and [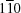] of an *a*-ZnO layer on *r*-sapphire on growth temperature

Temperature (K)	*R*_1_	*R*_2_
573	0.243 (3)	1.000 (1)
773	0.391 (3)	1.000 (1)
873	0.450 (3)	1.000 (1)
973	0.583 (5)	1.000 (1)
1073	0.660 (5)	1.000 (1)

## Appendix A Error analysis. General case

### A1. Equations

The number of measured parameters can be less than the number of sample parameters that a researcher would like to determine. Although the desired complete determination of the sample parameters is impossible in this case, some useful information can be retrieved from the experimental data. Below we show that, in general, the sample parameters can be divided into the following groups: (i) determined, (ii) partially determined and (iii) undetermined. The first type of parameters can be found from the provided experimental data with reasonable inaccuracies. The correct value of each parameter from the second group, considered separately, cannot be determined sufficiently accurately. However, there are some constraints on the set of ‘partially determined’ parameters imposed by the measurement results. This means that, fixing the values of some of these parameters on the basis of some other information (*e.g.* information concerning preparation of the sample), one can find the correct values of the other partially determined parameters. The ‘undetermined’ parameters are those for which the measured data provide no useful information.

If all the parameters are determined, equation (32) provides the estimated values of the parameters’ errors. However, if there are some ‘partially determined’ or ‘undetermined’ sample parameters, the Hessian is singular and the matrix  is not correctly defined. To resolve this problem one can consider an eigenbasis of the Hessian matrix and exclude eigenvectors corresponding to zero (or too small) eigenvalues, from determination of the errors. More precisely, the errors corresponding to such eigenvectors must be estimated from some conditions different from those of equation (28). Below it is shown that these conditions are provided by normalization of the parameters *x_j_*.

We slightly modify equation (27) by introducing the cost function error , independent of errors of the measured parameters *y*_β_:

This error accounts for calculation inaccuracies and is introduced to make the error evaluation procedure more stable.

Let the orthogonal matrix  describe the linear transformation of the sample parameters, diagonalizing the Hessian:

The solution provided by equation (31) is then simplified:

where

characterizes the influence of the finite calculation precision on the derivatives of the cost function.

Equation (35) is valid for nonzero eigenvalues only. Normalization of the parameters  implies that . Then one can assume that the following condition is satisfied:

Finally, from equations (35) and (37) one can find the following estimation for the mean squared error of the transformed sample parameters :

where

Then, the errors of the initial sample parameters can be calculated as

To complete the description of the error estimation protocol, one needs to assign a certain value to . This quantity was introduced to resolve the ambiguity arising when the cost function does not depend on some sample parameter (*e.g. x*_*n*_). Such a parameter corresponds to the zero eigenvalue of the Hessian: λ_*n*′_ = 0, *x*_*n*′_ = *x*_*n*_. On the other hand, one also has *Z*_*n*_ = 0 and, finally, , which means that introduction of nonzero quantity  enables us to detect undetermined parameters by large estimated errors (otherwise, the estimated error is indeterminate and can happen to have a small value). For correctly determined parameters, the introduction of  should not change the error estimation significantly: , where *N* is the number of the found sample parameters. Then, for all *j*′ the quantities  can be assigned the following value:

where in calculations one can take  (the value must be much less than the anticipated relative errors of the parameters but larger than the calculation errors caused by finite machine precision).

### A2. Parameters classification

The parameters for which equation (40) provides reasonably small errors (Δ*x_j_* < η ≃ 0.5) are classified as determined from the measurement results. The parameters that do not satisfy this condition can be either partially determined or undetermined. To distinguish between these two groups of sample parameters, we evaluate the constrained errors.

The above-described (unconstrained) error estimation was based on the assumption that all the sample parameters are initially unknown and can take any values (consistent with normalization). One can also make a constrained error estimation for each of the parameters, assuming that the considered parameter is the only unknown quantity, while the values of all other parameters are fixed. For a parameter with a large unconstrained error Δ*x_j_* > η, the constrained error can turn out to be small: Δ_c_ *x_j_* < η. In this case the parameter is found to be partially determined. If both the unconstrained and the constrained errors are large, the parameter is undetermined.

Similarly to the above-described calculations, the constrained error can be determined in the following way:

Summarizing, the procedure of error evaluation and parameter classification can be divided into the following steps:

(*a*) the unknown sample parameters are normalized, , and the cost function  is constructed;

(*b*) the optimal values , minimizing the cost function, are found;

(*c*) the Hessian  and the mixed derivatives matrix  are calculated using equations (29) and (30);

(*d*) the Hessian is diagonalized; transformation matrix  is constructed from normalized eigenvectors of the Hessian;

(*e*) unconstrained errors  are calculated on the basis of equations (38)–(41) [Disp-formula fd40] [Disp-formula fd41];

(*f*) constrained errors  are calculated on the basis of equation (42);

(*g*) the type is determined in the following way for each parameter:

(i) if , the parameter *x_j_* is determined;

(ii) if , , the parameter *x_j_* is partially determined;

(iii) if , , the parameter *x_j_* is undetermined.
